# From complex algorithms to clinical practice: a multicenter machine learning model and simplified decision tree for predicting cachexia risk in gastric cancer

**DOI:** 10.3389/fonc.2026.1767547

**Published:** 2026-03-10

**Authors:** Jian Zhao, Yu Deng, Yajie Guo, Yaoyao Wu, Xiaozhou Yang, Tengyu Zeng, Yihuan Qiao, Huadong Zhao, Jiawei Song, Beilei Hou, Qianyong Yang

**Affiliations:** 1 Department of Endocrinology, No 908th Hospital of Chinese PLA Joint Logistic Support Force, Nanchang, China; 2 Department of Digestive Surgery, Xijing Hospital of Digestive Diseases, Fourth Military Medical University, Xi’an, China; 3 State Key Laboratory of Holistic Integrative Management of Gastrointestinal Cancers and National Clinical Research Center for Digestive Diseases, Xijing Hospital of Digestive Diseases, Fourth Military Medical University, Xi’an, China; 4 Department of General Surgery, Tangdu Hospital, Air Force Medical University, Xi’an, China; 5 Department of General Surgery, No 908th Hospital of Chinese PLA Joint Logistic Support Force, Nanchang, China

**Keywords:** cachexia, decision tree, external validation, gastric cancer, machine learning, nutritional assessment, prediction model

## Abstract

**Background:**

Cachexia is a frequent, specific metabolic syndrome that severely compromises survival in gastric cancer (GC). While early diagnosis is paramount, existing screening methods are limited by complexity and suboptimal accuracy. There is an urgent need for an efficient, data-driven tool derived from routine clinical parameters.

**Methods:**

In this multicenter retrospective study, we analyzed data from three independent hospitals. Variable selection was performed using univariable and multivariable analyses. We constructed and compared multiple machine learning (ML) models to predict cachexia risk. The models’ discriminative ability, calibration, and clinical net benefit were comprehensively evaluated via AUC, calibration plots, and Decision Curve Analysis (DCA).

**Results:**

The study included 1,570 GC patients (cachexia prevalence: 30.3%). Patients were divided into training (n=920), internal testing (n=350), and external validation (n=300) cohorts. Cachexia was significantly associated with poor nutritional status, elevated inflammation, and inferior overall survival (P < 0.01). The Random Forest (RF) model yielded the best performance, maintaining excellent stability across the internal test set (AUC = 0.898) and external validation set (AUC = 0.913). To enhance clinical utility, we further derived a simplified decision tree model based on three accessible markers: CA19-9, CEA, and albumin. This simplified tool retained high diagnostic accuracy (AUC > 0.783) and demonstrated significant positive net benefits in DCA.

**Conclusion:**

We successfully established and externally validated a high-performance ML model for predicting GC-associated cachexia. Crucially, the derived simplified decision tree offers a convenient, highly generalizable tool for clinicians to identify high-risk patients using routine laboratory tests, enabling earlier precision nutritional management.

## Introduction

Gastric cancer (GC) remains one of the most common and lethal malignancies worldwide, with over one million new cases diagnosed annually (1). Most patients present at an advanced stage, posing substantial challenges for treatment and prognosis (2). Although multimodal strategies including surgery, chemotherapy, targeted therapy, and immunotherapy have improved clinical management, overall survival remains unsatisfactory (3–5).

Cachexia is a Common and debilitating complication in GC, characterized by progressive skeletal muscle loss driven by tumor-induced metabolic dysregulation (6, 7). Unlike simple malnutrition, cachexia involves complex interactions among pro-inflammatory cytokines, protein degradation pathways, appetite-regulating networks, and energy metabolism (8, 9). Its prevalence is striking, affecting 30–40% of newly diagnosed patients and an even greater proportion of those with advanced disease (10). Cachexia severely compromises quality of life, reduces treatment tolerance, increases surgical risks, and markedly shortens survival (11, 12). Once refractory, it is largely irreversible even with intensive nutritional support, underscoring the urgent need for early identification and intervention (13).

Growing evidence suggests that several composite laboratory indices can serve as effective proxies for the pathophysiological processes underlying cachexia. Among these, the Prognostic Nutritional Index (PNI), Systemic Immune-inflammation Index (SII), Neutrophil-to-Lymphocyte Ratio (NLR), and Creatinine-to-Cystatin C Ratio (CCR) are particularly relevant (14–17). These metrics collectively capture the interplay between systemic inflammation, immune dysfunction, and nutritional depletion (18). Their role in predicting cancer-related outcomes has been increasingly recognized, yet their integrated use for early cachexia risk stratification in GC remains underexplored.

The early and accurate diagnosis of cachexia poses a significant challenge. Existing diagnostic modalities, though diverse, are frequently time-consuming and labor-intensive, and their efficacy for early-stage detection remains suboptimal (19). Recently, machine learning (ML) has emerged as a promising approach for risk prediction in oncology, with the ability to model complex, nonlinear relationships in high-dimensional clinical data (20, 21). However, existing cachexia-related ML studies are limited by single-center designs, small sample sizes, and poor model interpretability, restricting their clinical utility.

Therefore, this study aims to establish cachexia prediction models based on a multicenter cohort of GC patients. Using routinely available clinical and laboratory indicators at first hospital admission, we applied multiple ML algorithms, performed rigorous internal and external validation, and derived interpretable clinical decision rules. Thereby enabling timely nutritional and metabolic interventions to improve clinical outcomes and survival.

## Methods

### Patients

This study was a multicenter, retrospective analysis based on data collected from three medical centers in China, Xijing Hospital (training cohort), Tangdu Hospital (testing cohort), and Nanchang Hospital (external validation cohort). Consecutive patients with pathologically confirmed GC admitted between January 2018 and December 2023 were enrolled. The inclusion criteria were as follows: (1) Age ≥ 18 years; (2) Histologically confirmed primary GC; (3) Availability of complete preoperative clinical records, laboratory tests, and imaging data; (4) Availability of complete follow-up data; (5) undergone gastric cancer resection surgery. The exclusion criteria were:(1) Presence of another primary malignancy;(2) Severe renal insufficiency (eGFR < 30 mL/min/1.73 m²), hepatic failure (Child class B or C), or heart failure (New York Heart Association class III or IV); (3) Acute gastrointestinal complications such as perforation; (4) History of autoimmune disease; (5) Patient with distant metastasis.

Clinical data were obtained through the electronic medical record systems of the participating hospitals, including demographic information, clinicopathological features, and laboratory results on the date of first admission. All patients were followed up regularly after surgery (**Supplementary Figure 1**). Follow-up data included overall survival, complication events, and the presence of cancer cachexia. Cachexia was diagnosed according to international consensus criteria: weight loss > 5% over the past six months (excluding cases caused by simple starvation, fluid retention, or other non-tumor-related factors); or BMI < 20 kg/m² with concomitant weight loss > 2%; or CT assessed skeletal muscle index consistent with sarcopenia plus weight loss > 2% (8). The study protocol was approved by the institutional review boards of all participating centers, with informed consent waived due to the retrospective nature of the study.

### Statistical analysis

All statistical analyses were performed using R software (version 5.1). A two-sided P-value < 0.05 was considered statistically significant. To minimize inter-center heterogeneity, a unified standardized preprocessing pipeline was applied across the three cohorts. Continuous variables were summarized as median (interquartile range) and compared using the rank-sum test. Categorical variables were presented as frequency and percentage and compared using the chi-square test or Fisher’s exact test. Survival curves were generated using the K-M method, and differences between groups were compared using the log-rank test. To reduce dimensionality and identify significant predictors, univariate analyses were first conducted to screen potential variables, followed by feature selection using least absolute shrinkage and selection operator (LASSO) regression. Multiple ML models were then developed using the “caret” package, including Random Forest, Gradient Boosting Machine (GBM), XGBoost, logistic regression (forward, backward, and stepwise selection), Elastic Net, and Support Vector Machine (SVM). Model discrimination was assessed using receiver operating characteristic (ROC) curves and the area under the curve (AUC). Calibration was evaluated using test. SHapley Additive exPlanations (SHAP) analysis was utilized to illustrate the contribution of each predictor and visualize their relative importance in the final model. The final models were validated in the training, testing, and external validation cohorts.

## Results

### Baseline characteristics

**Figure 1:**
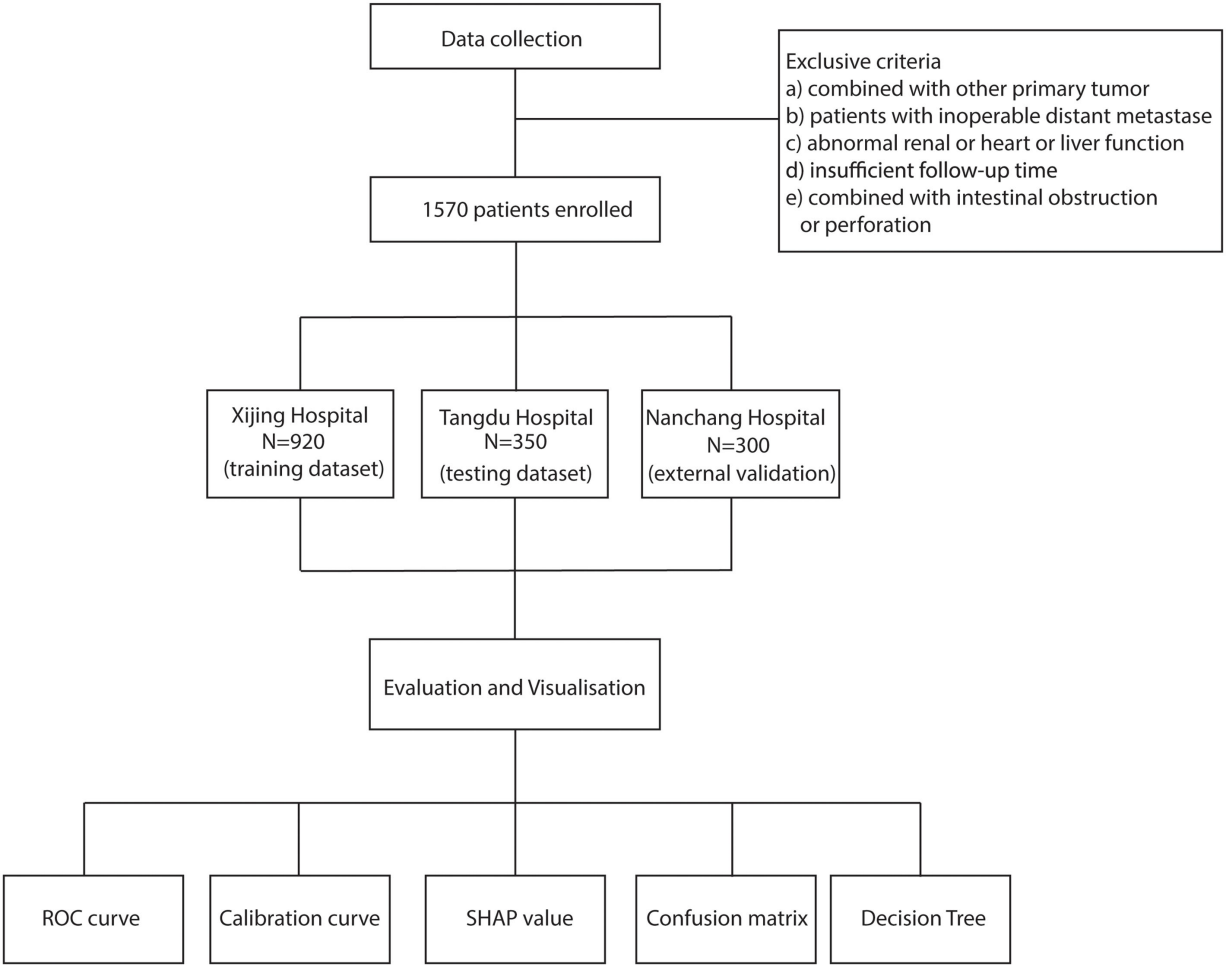
Study flowchart. The figure illustrates the overall study design and data distribution. A total of 1,570 patients with gastric cancer from three hospitals were included and divided into the training, testing, and external validation cohorts.

A total of 1,570 patients with GC admitted between January 2018 and December 2023 across three medical centers were included in this study (**Figure 1**). Patients were stratified into a training cohort (Xijing Hospital, n = 920), a testing cohort (Tangdu Hospital, n = 350), and an external validation cohort (Nanchang Hospital, n = 300). No significant differences in baseline clinical characteristics were observed across the three cohorts (**Table 1**). The overall cohort had a median age of 59.0 years, with males accounting for 75.6%. T stage III and IV disease was predominant (59.5%). The overall incidence of cachexia was 31.0%, with no significant differences among the three centers (P = 0.967). Other laboratory parameters, including inflammatory markers, nutritional indices, and tumor biomarkers, were also comparable between cohorts (all P > 0.05).

**Table 1:** Baseline characteristics of patients across the three participating centers.

Characteristic	[ALL] N=1570	XJ hospital N=920	TD hospital N=350	NC hospital N=300	p
cachexia:					0.567
0	1083 (69.0%)	644 (70.0%)	235 (67.1%)	204 (68.0%)	
1	487 (31.0%)	276 (30.0%)	115 (32.9%)	96 (32.0%)	
gender:					0.329
male	1187 (75.6%)	686 (74.6%)	275 (78.6%)	226 (75.3%)	
female	383 (24.4%)	234 (25.4%)	75 (21.4%)	74 (24.7%)	
age	59.0 [51.0;66.0]	59.0 [51.0;66.0]	59.0 [52.0;65.0]	58.0 [52.0;65.0]	0.617
BMI	22.7 [20.6;25.0]	22.7 [20.6;24.9]	22.7 [20.7;24.8]	22.6 [20.5;25.1]	0.866
T stage					0.579
1	400 (25.5%)	248 (27.0%)	83 (23.7%)	69 (23.0%)	
2	237 (15.1%)	135 (14.7%)	61 (17.4%)	41 (13.7%)	
3	359 (22.9%)	207 (22.5%)	78 (22.3%)	74 (24.7%)	
4	574 (36.6%)	330 (35.9%)	128 (36.6%)	116 (38.7%)	
N stage					0.739
0	672 (42.8%)	391 (42.5%)	158 (45.1%)	123 (41.0%)	
1	256 (16.3%)	146 (15.9%)	62 (17.7%)	48 (16.0%)	
2	228 (14.5%)	139 (15.1%)	47 (13.4%)	42 (14.0%)	
3	414 (26.4%)	244 (26.5%)	83 (23.7%)	87 (29.0%)	
albumin	36.8 [32.7;41.5]	36.7 [32.6;41.4]	37.5 [32.7;42.2]	36.0 [33.1;41.3]	0.394
immunoglobulin	23.8 [20.4;26.9]	23.6 [20.4;26.9]	24.2 [20.7;27.1]	23.8 [20.3;26.7]	0.371
DB	4.50 [3.30;6.30]	4.50 [3.30;6.40]	4.50 [3.50;6.18]	4.30 [3.30;6.12]	0.409
UDB	6.90 [4.80;9.90]	6.70 [4.77;10.0]	7.30 [5.30;9.78]	6.80 [4.60;9.45]	0.17
AKP	69.0 [57.0;85.0]	69.0 [57.0;85.0]	70.0 [60.0;84.0]	69.5 [58.0;86.0]	0.676
rGABA	16.0 [11.0;24.0]	16.0 [11.0;24.0]	16.0 [12.0;23.0]	15.0 [11.0;22.2]	0.231
ALT	31.0 [17.0;53.0]	31.0 [17.0;53.0]	30.0 [17.0;54.0]	31.0 [16.0;53.0]	0.789
AST	32.0 [18.0;56.0]	32.0 [18.0;57.0]	31.5 [18.0;53.8]	32.0 [19.0;59.2]	0.57
cholesterol	4.14 [3.59;4.80]	4.14 [3.59;4.79]	4.13 [3.64;4.82]	4.14 [3.53;4.74]	0.981
TBA	3.00 [1.80;5.60]	2.96 [1.73;5.50]	3.08 [1.95;6.06]	3.04 [1.90;5.48]	0.46
TG	1.17 [0.90;1.57]	1.16 [0.88;1.55]	1.18 [0.95;1.57]	1.19 [0.88;1.57]	0.701
HDL	1.12 [0.95;1.31]	1.12 [0.95;1.33]	1.12 [0.96;1.29]	1.12 [0.93;1.33]	0.961
LDL	2.48 [2.04;2.92]	2.49 [2.03;2.93]	2.43 [2.08;2.93]	2.50 [2.02;2.91]	0.966
Urea	5.04 [4.16;6.05]	5.04 [4.22;6.01]	5.04 [4.07;6.06]	5.08 [4.06;6.31]	0.963
Uric acid	246 [197;300]	246 [198;299]	246 [200;305]	240 [196;294]	0.597
Crine	95.0 [85.0;105]	95.0 [84.0;105]	96.0 [85.0;106]	97.0 [85.0;104]	0.311
GLU	5.62 [5.05;7.53]	5.61 [5.02;7.63]	5.68 [5.10;7.42]	5.60 [5.05;7.20]	0.738
Na	141 [139;143]	141 [139;143]	141 [139;143]	141 [139;143]	0.586
K	4.14 [3.89;4.40]	4.14 [3.89;4.39]	4.16 [3.90;4.39]	4.11 [3.87;4.41]	0.727
Cl	105 [102;107]	105 [102;107]	105 [102;107]	104 [102;107]	0.929
Ca	2.04 [1.92;2.17]	2.04 [1.91;2.17]	2.06 [1.94;2.19]	2.02 [1.93;2.15]	0.076
AFP	2.73 [1.97;3.90]	2.72 [1.97;3.90]	2.80 [2.04;3.95]	2.71 [1.89;3.82]	0.389
CEA	2.27 [1.38;3.88]	2.26 [1.36;3.78]	2.29 [1.47;4.30]	2.28 [1.45;4.12]	0.342
CA19-9	11.3 [6.29;25.3]	10.9 [6.23;24.4]	12.3 [5.91;29.5]	12.0 [6.87;25.6]	0.457
CA125	11.4 [8.24;16.4]	11.3 [8.22;15.9]	11.3 [7.83;16.6]	11.5 [8.75;17.9]	0.252
WBC	8.02 [5.75;11.2]	8.07 [5.69;11.2]	7.90 [5.80;11.1]	7.85 [5.92;11.0]	0.967
Neuro R	0.86 [0.65;0.90]	0.86 [0.65;0.91]	0.85 [0.63;0.90]	0.86 [0.65;0.90]	0.311
Lymp R	0.10 [0.06;0.25]	0.10 [0.06;0.25]	0.10 [0.06;0.27]	0.11 [0.07;0.25]	0.679
RBC	4.32 [3.88;4.79]	4.32 [3.86;4.79]	4.33 [3.93;4.83]	4.32 [3.89;4.72]	0.862
hemoglobin	129 [110;146]	129 [110;146]	131 [110;148]	126 [109;142]	0.11
HCT	0.39 [0.34;0.44]	0.39 [0.34;0.43]	0.40 [0.35;0.44]	0.39 [0.34;0.43]	0.166
PLT	199 [154;249]	199 [154;249]	200 [157;252]	202 [157;247]	0.826
D_2	310 [52.5;698]	310 [39.8;665]	290 [3.85;660]	340 [158;880]	0.189
APTT	34.6 [30.5;37.7]	34.6 [30.5;37.7]	34.2 [29.8;37.6]	35.0 [30.9;37.9]	0.309
PT	13.0 [12.1;13.6]	13.0 [12.1;13.6]	12.9 [12.0;13.6]	13.1 [12.4;13.7]	0.171
SII	1434[551;2701]	1439[532;2729]	1503[532;2719]	1351 [642;2463]	0.92
PNI	41.6 [36.6;48.9]	41.5 [36.2;48.7]	42.0 [36.9;49.4]	41.8 [37.1;48.6]	0.555
NLR	8.34 [2.52;14.4]	8.37 [2.60;14.9]	8.40 [2.34;14.3]	7.99 [2.58;13.4]	0.653
CCR	120 [106;142]	120 [106;143]	118 [104;136]	125 [108;142]	0.046

DB, Direct Bilirubin; UDB, Indirect Bilirubin; AKP, Alkaline Phosphatase; rGABA, Reduced Gamma-Aminobutyric Acid; ALT, Alanine Aminotransferase; AST, Aspartate Aminotransferase; cholesterol, Total Cholesterol; TBA, Total Bile Acids; TG, Triglycerides; HDL, High-Density Lipoprotein; LDL, Low-Density Lipoprotein; Urea, Urea Nitrogen; uric_acid, Uric Acid; Crine, Creatinine; GLU, Glucose; Na, Sodium; K, Potassium; Cl, Chloride; Ca2, Calcium; AFP, Alpha-fetoprotein; CEA, Carcinoembryonic Antigen; CA19-9, Carbohydrate Antigen 19-9; CA125, Carbohydrate Antigen 125; WBC, White Blood Cell Count; Neuro R, Neutrophil Ratio; Lymp R, Lymphocyte Ratio; RBC, Red Blood Cell Count; hemoglobin, Hemoglobin; HCT, Hematocrit; PLT, Platelet Count; D_2, D-dimer; APTT, Activated Partial Thromboplastin Time; PT, Prothrombin Time; SII, Systemic Immune-inflammation Index; PNI, Prognostic Nutritional Index; NLR, Neutrophil-to-Lymphocyte Ratio; CCR, Creatinine-to-Cystatin C Ratio.

### Comparison of cachexia-related parameters

Baseline characteristics were further compared between patients with cachexia (n = 487) and those without (n = 1,083). Patients with cachexia had significantly worse nutritional status, as reflected by lower BMI, albumin, and hemoglobin levels (all P < 0.001). In addition, systemic inflammatory markers showed marked differences between groups. Patients with cachexia had significantly higher systemic SII and NLR, and lower PNI level compared with non-cachexia patients (all P < 0.001), whereas no significant difference was found for CCR (**Table 2**).

**Table 2:** Comparison of clinical and laboratory parameters between cachexia and non-cachexia groups.

Characteristic	Non-cachexia N=1083	Cachexia N=487	p
gender:			0.269
male	828 (76.5%)	359 (73.7%)	
female	255 (23.5%)	128 (26.3%)	
age	59.0 [51.0;65.0]	59.0 [52.0;66.0]	0.269
BMI	23.5 [21.9;25.5]	20.8 [19.2;22.5]	<0.001
T stage			<0.001
1	379 (35.0%)	21 (4.31%)	
2	205 (18.9%)	32 (6.57%)	
3	265 (24.5%)	94 (19.3%)	
4	234 (21.6%)	340 (69.8%)	
N stage			<0.001
0	585 (54.0%)	87 (17.9%)	
1	198 (18.3%)	58 (11.9%)	
2	134 (12.4%)	94 (19.3%)	
3	166 (15.3%)	248 (50.9%)	
NRS2002:			<0.001
0	367 (33.9%)	103 (21.1%)	
1	716 (66.1%)	384 (78.9%)	
albumin	38.0 [33.5;42.5]	34.7 [30.8;39.0]	<0.001
immunoglobulin	24.1 [20.9;27.4]	23.0 [19.6;26.4]	<0.001
DB	4.60 [3.40;6.40]	4.30 [3.20;5.95]	0.008
UDB	7.30 [5.20;10.4]	6.00 [4.10;8.70]	<0.001
AKP	71.0 [59.0;87.0]	66.0 [53.0;82.5]	<0.001
rGABA	17.0 [12.0;25.0]	14.0 [10.0;22.0]	<0.001
ALT	30.0 [17.0;54.5]	31.0 [17.5;50.5]	0.848
AST	30.0 [18.0;54.5]	35.0 [19.0;60.0]	0.011
cholesterol	4.14 [3.60;4.85]	4.13 [3.57;4.71]	0.156
TBA	2.90 [1.78;5.32]	3.18 [1.90;6.53]	0.006
TG	1.20 [0.90;1.67]	1.14 [0.89;1.42]	0.001
HDL	1.11 [0.96;1.31]	1.14 [0.92;1.34]	0.666
LDL	2.49 [2.06;2.92]	2.45 [2.01;2.92]	0.154
Urea	5.08 [4.19;6.02]	5.01 [4.14;6.24]	0.977
uric_acid	250 [204;303]	233 [188;292]	<0.001
Crine	96.0 [85.0;106]	93.0 [84.0;103]	0.012
GLU	5.59 [5.02;7.16]	5.72 [5.11;7.90]	0.057
Na	141 [139;143]	140 [138;142]	<0.001
K	4.14 [3.88;4.38]	4.15 [3.89;4.44]	0.089
Cl	105 [102;107]	104 [102;107]	0.358
CysC	0.79 [0.68;0.89]	0.76 [0.64;0.89]	0.035
Ca2	2.07 [1.94;2.19]	2.00 [1.88;2.12]	<0.001
AFP	2.72 [1.96;3.84]	2.75 [2.00;4.24]	0.11
CEA	2.05 [1.28;3.16]	2.95 [1.78;7.69]	<0.001
CA19-9	9.24 [5.53;15.2]	34.6 [11.6;54.6]	<0.001
CA125	10.7 [7.90;14.8]	13.4 [9.19;21.0]	<0.001
WBC	7.75 [5.67;11.0]	8.56 [6.04;11.5]	0.048
Neuro_R	0.84 [0.63;0.90]	0.88 [0.75;0.91]	<0.001
Lymp_R	0.12 [0.06;0.28]	0.09 [0.06;0.17]	<0.001
RBC	4.42 [3.94;4.87]	4.18 [3.81;4.56]	<0.001
hemoglobin	132 [114;148]	119 [105;138]	<0.001
HCT	0.40 [0.35;0.44]	0.37 [0.33;0.41]	<0.001
PLT	198 [157;246]	201 [152;256]	0.231
D_2	290 [5.74;635]	370 [170;1000]	<0.001
APTT	34.6 [30.3;37.5]	34.6 [31.4;38.3]	0.067
PT	12.9 [12.0;13.6]	13.2 [12.4;13.8]	<0.001
SII	1179 [465;2562]	1816 [868;2900]	<0.001
PNI	43.2 [37.8;50.2]	38.4 [34.4;44.8]	<0.001
NLR	6.76 [2.31;14.3]	9.72 [4.17;14.7]	<0.001
CCR	120 [106;140]	122 [105;143]	0.638

DB, Direct Bilirubin; UDB, Indirect Bilirubin; AKP, Alkaline Phosphatase; rGABA, Reduced Gamma-Aminobutyric Acid; ALT, Alanine Aminotransferase; AST, Aspartate Aminotransferase; cholesterol, Total Cholesterol; TBA, Total Bile Acids; TG, Triglycerides; HDL, High-Density Lipoprotein; LDL, Low-Density Lipoprotein; Urea, Urea Nitrogen; uric_acid, Uric Acid; CysC, Cystatin C; GLU, Glucose; Na, Sodium; K, Potassium; Cl, Chloride; Ca2, Calcium; AFP, Alpha-fetoprotein; CEA, Carcinoembryonic Antigen; CA19-9, Carbohydrate Antigen 19-9; CA125, Carbohydrate Antigen 125; WBC, White Blood Cell Count; Neuro_R, Neutrophil Ratio; Lymp_R, Lymphocyte Ratio; RBC, Red Blood Cell Count; hemoglobin, Hemoglobin; HCT, Hematocrit; PLT, Platelet Count; D_2, D-dimer; APTT, Activated Partial Thromboplastin Time; PT, Prothrombin Time; SII, Systemic Immune-inflammation Index; PNI, Prognostic Nutritional Index; NLR, Neutrophil-to-Lymphocyte Ratio; CCR, Creatinine-to-Cystatin C Ratio..

Subgroup analyses by medical center confirmed the robustness and generalizability of these findings. Across all three centers, cachexia patients consistently demonstrated poorer nutritional indicators. Specifically, BMI, serum albumin and PNI were significantly lower, while SII was consistently elevated in cachexia patients (all P < 0.01). NLR was also higher in cachexia patients across most centers, except at Tangdu Hospital where no significant difference was observed. Tumor markers including carcinoembryonic antigen (CEA), carbohydrate antigen 19-9 (CA19-9) and carbohydrate antigen 125 (CA125) were significantly elevated in cachexia patients across all three centers (all P < 0.01, **Supplementary Table 1**).

**Figure 2:**
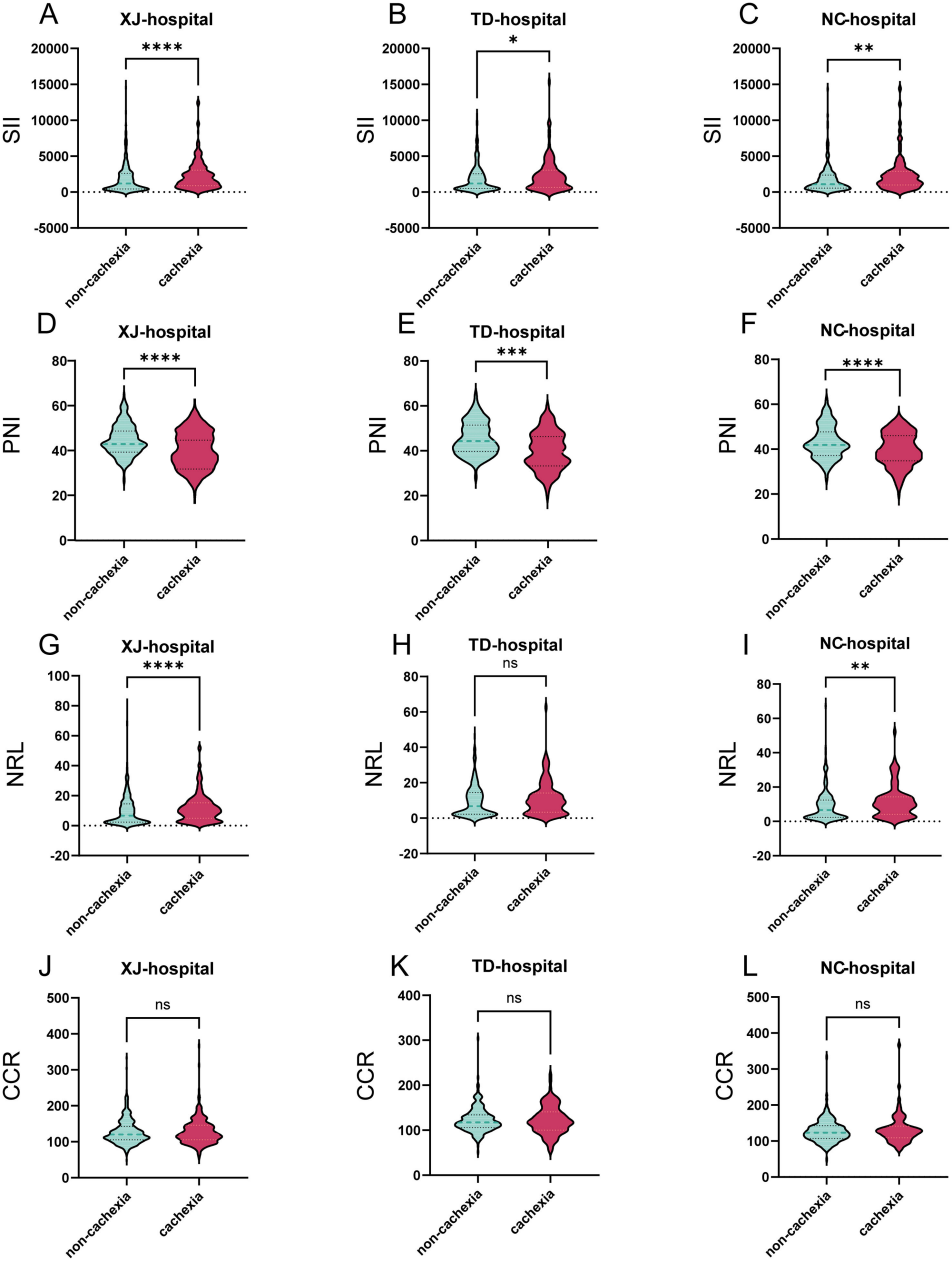
Violin plots comparing key variable distributions across three hospitals. **(A-C)** Distributions of the Systemic Immune-Inflammation Index (SII) between cachexia and non-cachexia patients in the Xijing, Tangdu, and Nanchang cohorts, respectively; **(D-F)** Distributions of the Prognostic Nutritional Index (PNI) across the three centers; **(G-I)** Distributions of the Neutrophil-to-Lymphocyte Ratio (NLR) between cachexia and non-cachexia patients across the three hospitals; **(J-L)** Distributions of the Creatinine-to-Cystatin C Ratio (CCR) in the three cohorts. The symbol of ns, *, **, *** and **** mean no significance, P < 0.05, P < 0.01, P < 0.001 and P < 0.0001, respectively.

Analysis of inflammatory and nutritional parameters further revealed systematic differences: cachexia patients exhibited markedly higher SII and NLR, while lower PNI levels than non-cachexia patients (**Figures 2A–I**). No significant differences were observed for CCR across these three centers (**Figures 2J–L**).

### Association between cachexia and long-term survival

**Figure 3:**
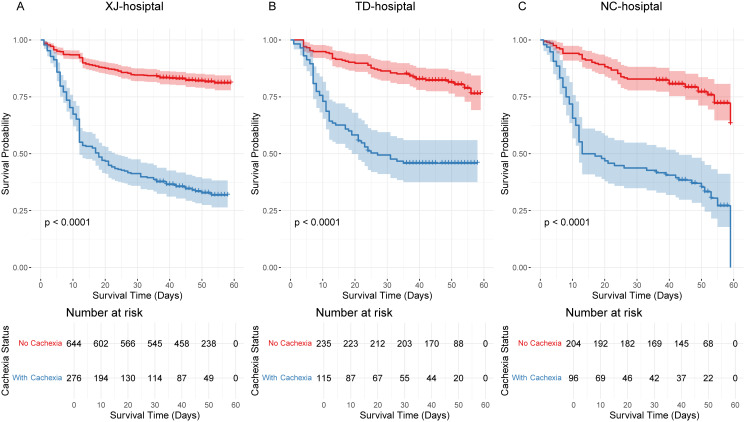
Kaplan–Meier survival curves comparing cachexia and non-cachexia groups. **(A)** Training cohort (Xijing Hospital), showing significantly shorter median survival in the cachexia group (P < 0.0001); **(B)** Testing cohort (Tangdu Hospital), consistent with the training cohort results (P < 0.0001); **(C)** External validation cohort (Nanchang Hospital), confirming a strong association between cachexia and poor prognosis (P < 0.0001).

Kaplan–Meier survival analyses were conducted to evaluate the prognostic impact of cachexia in GC patients across the three cohorts. Consistently, patients with cachexia exhibited significantly poorer survival compared to those without cachexia (all P < 0.0001). At Xijing Hospital, Tangdu Hospital, and Nanchang Hospital, survival probabilities were uniformly lower in the cachexia groups (**Figures 3A–C**, all P < 0.0001).

### Identification of independent predictors and machine learning model development

Univariate logistic regression analysis identified multiple clinical variables associated with cachexia (P < 0.05), including BMI, serum albumin, PNI, inflammatory indices (SII, NLR), and tumor markers (CEA, CA19-9) (**Supplementary Table 2**).

Variables with P < 0.1 in univariate analysis were entered into multivariate logistic regression. Independent predictors of cachexia included lower BMI, advanced T stage, lymph node metastasis (N3), elevated AFP, CEA, and CA19–9 levels. Interestingly, higher red blood cell (RBC) counts emerged as a protective factor, whereas composite inflammatory markers (SII, NLR, PNI) lost their significance in multivariate models (**Supplementary Table 3**). We found that the composite inflammatory indices exhibited severe multicollinearity, with VIF values of 21.90 for PNI, 10.09 for NLR, and 10.39 for SII. All these values are substantially above the conventional threshold of 10, indicating that the predictive information contained within these indices is highly redundant with other predictors in the model, particularly nutritional indicators (**Supplementary Table 4**).

**Figure 4:**
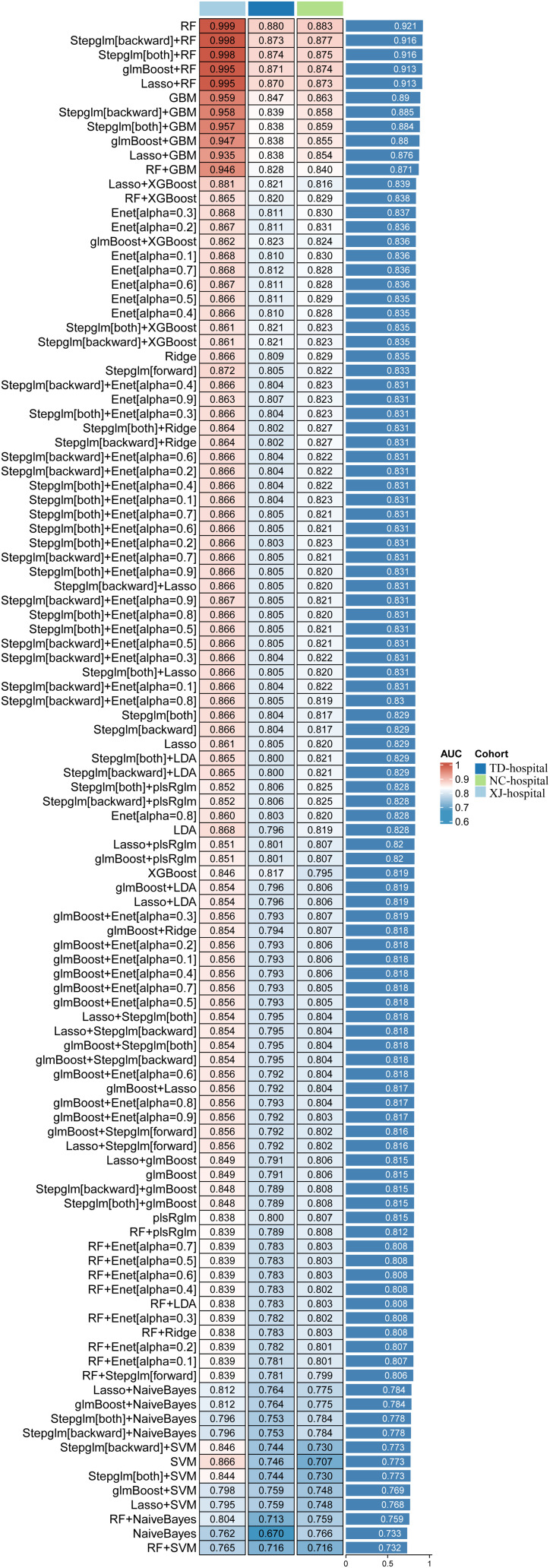
Model performance ranking. A systematic comparison of over 100 combinations of feature selection and machine learning algorithms was performed across the training, testing, and external validation cohorts. The optimal modeling strategy was selected based on the highest area under the ROC curve (AUC).

Based on these predictors, multiple ML models were developed and validated across cohorts. As shown in **Figure 4**, ensemble tree-based models consistently outperformed linear models, where Random Forest (RF) achieved the best performance. Gradient Boosting Machine (GBM) and stacking models (RF+GBM) also showed robust performance (AUC > 0.84). In contrast, linear models such as Lasso, Ridge, Elastic Net, and Linear Discriminant Analysis (LDA) yielded relatively modest performance (AUC 0.78–0.84). The RF model was ultimately selected as the optimal predictive model.

### Model validation, interpretability, and clinical decision rules

**Figure 5:**
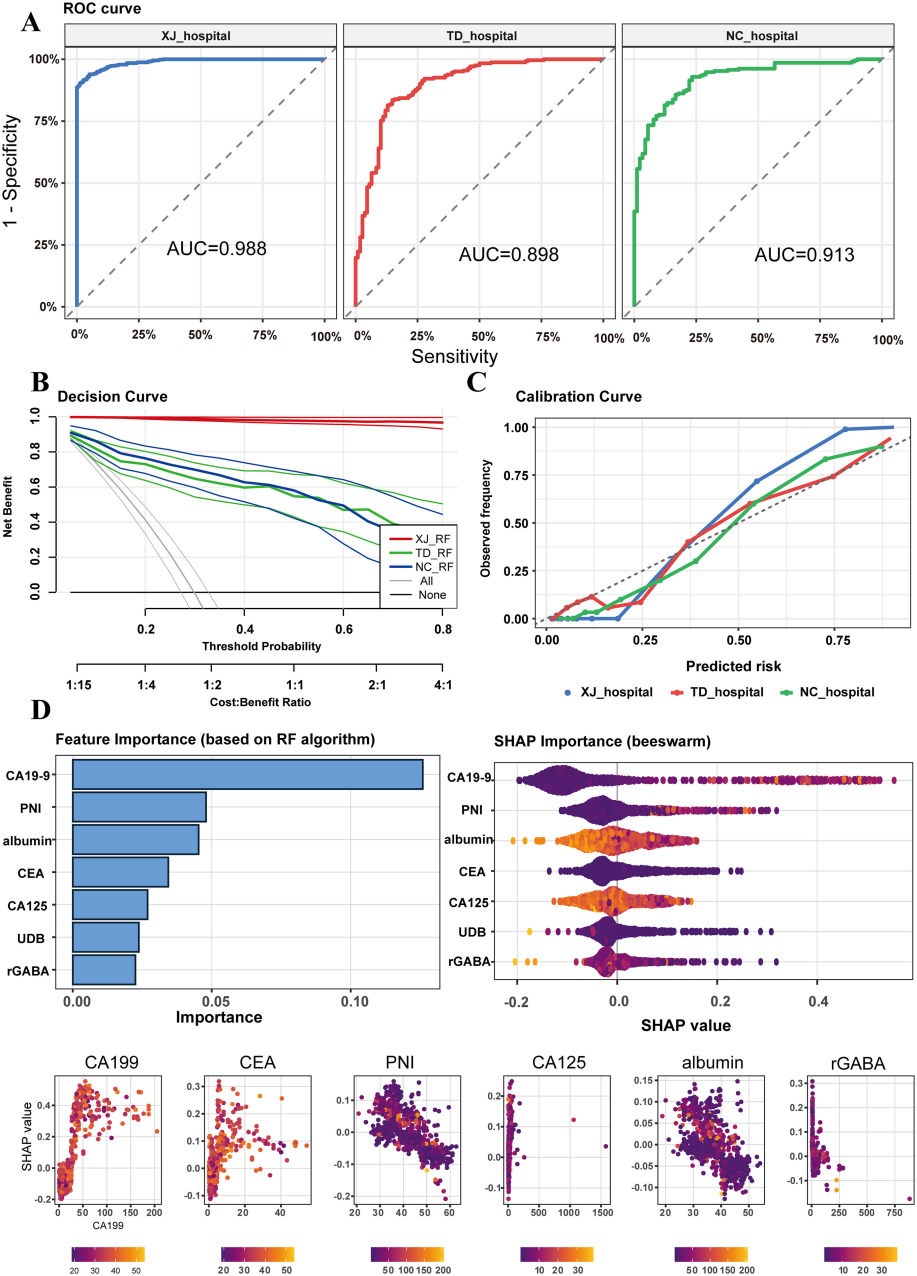
Validation of the optimal model across three hospitals. **(A)** ROC curves of the optimal Random Forest (RF) model in the three centers, with AUCs of 0.988, 0.898, and 0.913, respectively; **(B)** Decision curve analysis (DCA) demonstrating positive net clinical benefit across a wide range of threshold probabilities, with well-overlapping curves among the three centers; **(C)** Calibration plots (binned) showing high agreement between predicted and observed probabilities, indicating good model calibration; **(D)** SHAP value ranking of variables contributing most to model prediction, including CA19-9, CEA, PNI, albumin, CA125, and GABA.

The predictive performance of the RF model was comprehensively evaluated using ROC curves, decision curve analysis (DCA), and feature importance rankings. The model demonstrated excellent discrimination across all cohorts, with an AUC of 0.988 in the training cohort, and maintained strong generalization in the testing (AUC = 0.898) and external validation (AUC = 0.913) cohorts (**Figure 5A**).

DCA curves demonstrated net clinical benefit across all centers, outperforming “treat all” or “treat none” strategies (**Figure 5B**). Calibration plots indicated excellent agreement between predicted and observed probabilities, particularly in moderate-to-high risk ranges (**Figure 5C**). Feature importance analysis identified CA19-9, PNI, albumin, CEA, CA125, UDB and rGABA as the most influential predictors (**Figure 5D**).

**Figure 6:**
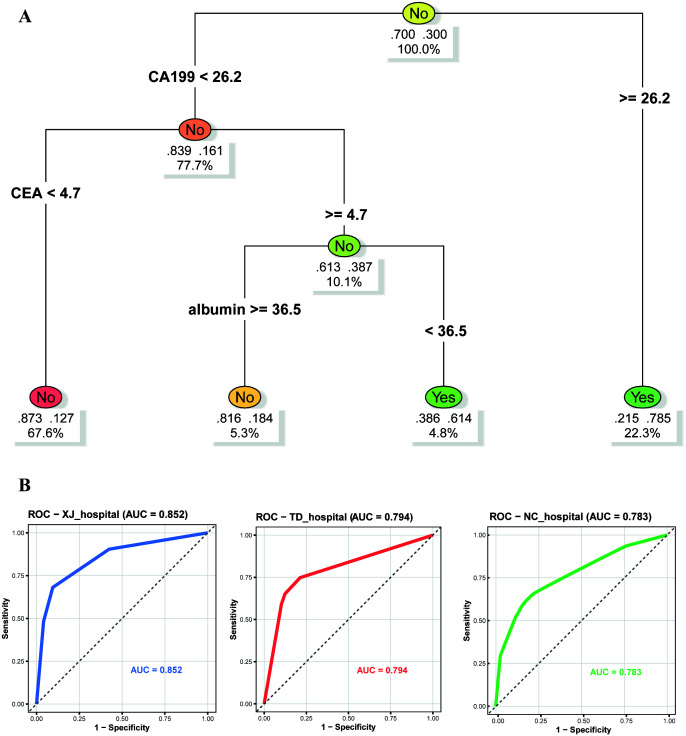
Visualization of the decision tree. **(A)** The primary split is based on CA19-9 < 26.2 U/mL, followed by further subdivisions according to CEA and albumin levels; **(B)** ROC curves of the decision tree model among the three hospitals.

For clinical ease of use, we constructed a decision tree based on CA19-9, CEA, and albumin (**Figure 6A**). With CA19-9 (26.2 U/mL) as the primary split, the model identifies low-risk (16.1%) and high-risk patients. The highest risk of cachexia (78.5%) was observed in patients with elevated CA19-9, elevated CEA (>4.7 ng/mL), and low albumin (<36.5 g/L). The model showed stable performance across centers, with AUCs of 0.852 (training), 0.794 (testing), and 0.783 (external validation). The closely aligned ROC curves (**Figure 6B**) confirm its strong generalizability as a bedside tool for surgical oncology.

## Discussion

In this multicenter study, we developed and validated a ML model to predict the preoperative risk of cachexia in patients with GC. A simplified decision tree model was further derived using three routinely available clinical indicators, namely serum albumin, CEA, and CA19-9. This tool demonstrated favorable accuracy and interpretability, with stable performance across internal and external validation cohorts, highlighting its potential for clinical application.

Patients with cachexia were more likely to present with significantly lower BMI, serum albumin, and hemoglobin levels. These findings are in line with previous reports (11). Cachexia is essentially a tumor-driven metabolic disorder. As tumor burden increases in advanced disease, the secretion of pro-inflammatory cytokines such as TNF-α and IL-6 is enhanced. This process activates the ubiquitin–proteasome system and the autophagy–lysosome pathway, leading to intensified skeletal muscle catabolism (22). Our study further demonstrated that systemic inflammatory indices, including SII, NLR, were significantly elevated in patients with cachexia across all centers (23, 24). This observation emphasizes the central role of systemic inflammation in the pathophysiology of cachexia (25, 26). However, these composite indices were not retained as independent predictors in multivariate analysis, likely due to collinearity with nutritional indicators, which illustrates the multifactorial and interactive nature of cachexia.

From the methodological perspective, tree-based ensemble models, especially RF, showed significantly better predictive performance than linear approaches such as logistic regression or Elastic Net (27). This is expected, because cachexia arises from complex nonlinear interactions among multiple factors. RF models integrate numerous decision trees, allowing them to capture such complex patterns and achieve higher predictive accuracy (28, 29). Our RF model maintained high and stable AUC values in the training, testing, and external validation cohorts, all above 0.91. Decision curve analysis further confirmed its clinical net benefit, indicating reliable generalizability across diverse patient populations.

To improve interpretability and enhance clinical translation, we constructed a simplified decision tree based on three key predictors. This model provided a clear stepwise stratification: patients with elevated CA19–9 were further differentiated by CEA levels, and finally stratified by serum albumin concentration. Its strength lies in transforming a complex black-box model into a transparent decision process that clinicians can easily apply in practice. Serum albumin emerged as a critical factor reflecting both nutritional status and systemic inflammation, while CA19–9 and CEA captured tumor burden and aggressiveness, confirming the tumor-driven nature of cachexia (30).

Elevated CA19–9 not only reflects tumor load but may also indicate aggressive tumor biology (31). Such tumors can secrete higher levels of pro-inflammatory cytokines including IL-6 and TNF-α, as well as cachexia-inducing factors such as LMF and PIF, which directly promote catabolism of muscle and adipose tissue (32, 33). Elevated CEA is also associated with advanced disease and poor outcomes (34). Recent evidence suggests that CEA may modulate immune cell function, intensifying tumor-associated immunosuppression and chronic inflammation (35). Persistent inflammation activates proteolytic systems and inhibits anabolic signaling such as the mTOR pathway, resulting in decreased protein synthesis and enhanced muscle breakdown (36). Serum albumin levels reflect both nutritional intake and systemic inflammation. Hypoalbuminemia has consistently been recognized as a strong independent predictor of cachexia (37). systemic inflammation increases vascular permeability and shifts hepatic protein synthesis toward acute-phase reactants such as C-reactive protein, while reduced intake due to anorexia worsens malnutrition (38, 39).

The major advantage of this study is that it bridges advanced ML with a simple and visualizable clinical decision tool. The decision tree relies only on three routine laboratory tests, which makes it particularly suitable for application in primary or resource-limited hospitals. This tool may facilitate preoperative screening and early nutritional interventions for GC patients at risk of cachexia.

Several limitations of this study should be acknowledged. First, the Random Forest algorithm exhibited inherent “model optimism” in the internal cohort (Xijing hospital: AUC 0.988, slope 2.106). Despite our efforts to penalize complexity via hyperparameter tuning, some overfitting to center-specific features occurred. However, the consistently robust performance in the independent external cohorts (Tangdu hospital: AUC 0.898, intercept 0.006; NanChang hospital: AUC 0.913) confirms that the model’s core predictive power is reliable and generalizable across different clinical settings. Second, as a retrospective study, selection bias and the heterogeneity of surgical management across the three centers cannot be entirely excluded. Third, the model was constructed using preoperative clinical indicators and lacks more granular assessments. Specifically, the exclusion of CT-based sarcopenia evaluation, which is a critical prognostic factor in gastrointestinal and liver oncology, as well as emerging biomarkers such as cytokines or metabolites, may have limited the model’s further refinement. Fourth, our analysis was based on static baseline data. In the clinical course of tumor progression, dynamic parameters—such as longitudinal weight changes, treatment response, or fluctuating inflammatory markers—often provide more nuanced prognostic value. Future prospective studies incorporating multi-omics and longitudinal data are warranted to enhance the precision and dynamic monitoring capabilities of this predictive tool.

## Conclusion

In summary, we established and validated a multicenter ML model that accurately predicts the risk of cachexia in GC patients. More importantly, we derived a simplified decision tree using three routine laboratory parameters, which combines strong predictive performance with excellent interpretability. This practical tool may enable clinicians to identify high-risk patients early, guide nutritional and metabolic interventions, and ultimately improve treatment outcomes and quality of life.

## Data Availability

The original contributions presented in the study are included in the article/**Supplementary Material**. Further inquiries can be directed to the corresponding authors.
