# Supplementary figures and images for: From complex algorithms to clinical practice: a multicenter machine learning model and simplified decision tree for predicting cachexia risk in gastric cancer
Source: Front Oncol. 2026 Mar 10;16:1767547. doi: 10.3389/fonc.2026.1767547 (PMC13008652; doi:10.3389/fonc.2026.1767547)

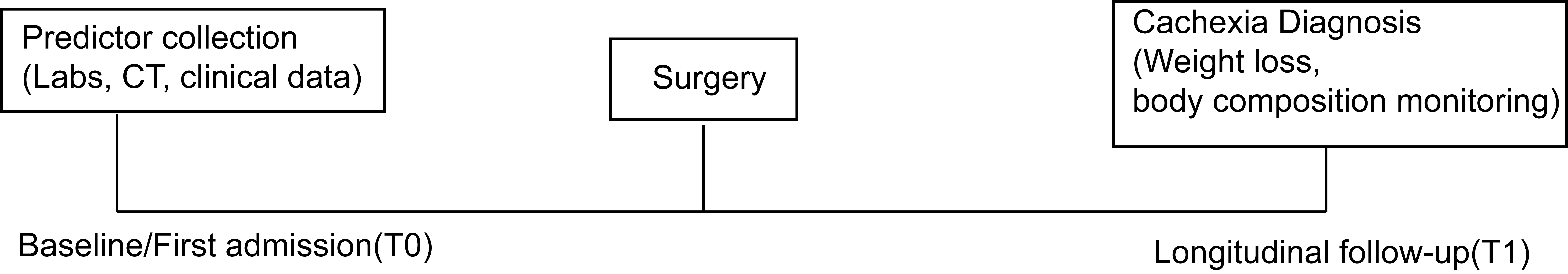

Supplement: Supplementary file 1 [file Image1.tif]
