# Supplementary material for: From complex algorithms to clinical practice: a multicenter machine learning model and simplified decision tree for predicting cachexia risk in gastric cancer
Source: Front Oncol. 2026 Mar 10;16:1767547. doi: 10.3389/fonc.2026.1767547 (PMC13008652; doi:10.3389/fonc.2026.1767547)
Supplement: Supplementary file 2 [file Table1.docx]

Table S1. Subgroup analysis of cachexia-related variables across medical centers.

| **characteristic** | **XJ_hospital** | | | **TD_hospital** | | | **NC_hospital** | | |
| --- | --- | --- | --- | --- | --- | --- | --- | --- | --- |
|  | **non-cachexia *N=644*** | **Cachexia *N=276*** | **p** | **non-cachexia *N=235*** | **Cachexia *N=115*** | **p** | **non-cachexia *N=204*** | **Cachexia *N=96*** | **p** |
| gender: |  |  | 0.298 |  |  | 0.968 |  |  | 0.418 |
| male | 487 (75.6%) | 199 (72.1%) |  | 184 (78.3%) | 91 (79.1%) |  | 157 (77.0%) | 69 (71.9%) |  |
| female | 157 (24.4%) | 77 (27.9%) |  | 51 (21.7%) | 24 (20.9%) |  | 47 (23.0%) | 27 (28.1%) |  |
| age | 58.0 [50.0;66.0] | 60.0 [52.0;66.0] | 0.203 | 59.0 [52.0;65.0] | 60.0 [53.0;67.5] | 0.116 | 58.5 [53.0;66.0] | 58.0 [48.8;64.0] | 0.145 |
| BMI | 23.5 [21.9;25.5] | 20.7 [19.0;22.3] | <0.001 | 23.6 [21.8;25.4] | 20.8 [19.6;23.0] | <0.001 | 23.6 (2.84) | 21.0 (3.11) | <0.001 |
| T stage |  |  | <0.001 |  |  | <0.001 |  |  | <0.001 |
| 1 | 234 (36.3%) | 14 (5.07%) |  | 79 (33.6%) | 4 (3.48%) |  | 66 (32.4%) | 3 (3.12%) |  |
| 2 | 121 (18.8%) | 14 (5.07%) |  | 50 (21.3%) | 11 (9.57%) |  | 34 (16.7%) | 7 (7.29%) |  |
| 3 | 155 (24.1%) | 52 (18.8%) |  | 55 (23.4%) | 23 (20.0%) |  | 55 (27.0%) | 19 (19.8%) |  |
| 4 | 134 (20.8%) | 196 (71.0%) |  | 51 (21.7%) | 77 (67.0%) |  | 49 (24.0%) | 67 (69.8%) |  |
| N stage |  |  | <0.001 |  |  | <0.001 |  |  | <0.001 |
| 0 | 342 (53.1%) | 49 (17.8%) |  | 131 (55.7%) | 27 (23.5%) |  | 112 (54.9%) | 11 (11.5%) |  |
| 1 | 118 (18.3%) | 28 (10.1%) |  | 43 (18.3%) | 19 (16.5%) |  | 37 (18.1%) | 11 (11.5%) |  |
| 2 | 82 (12.7%) | 57 (20.7%) |  | 28 (11.9%) | 19 (16.5%) |  | 24 (11.8%) | 18 (18.8%) |  |
| 3 | 102 (15.8%) | 142 (51.4%) |  | 33 (14.0%) | 50 (43.5%) |  | 31 (15.2%) | 56 (58.3%) |  |
| NRS2002: |  |  | <0.001 |  |  | 0.001 |  |  | 0.422 |
| 0 | 224 (34.8%) | 60 (21.7%) |  | 88 (37.4%) | 22 (19.1%) |  | 55 (27.0%) | 21 (21.9%) |  |
| 1 | 420 (65.2%) | 216 (78.3%) |  | 147 (62.6%) | 93 (80.9%) |  | 149 (73.0%) | 75 (78.1%) |  |
| albumin | 38.0 [33.2;42.7] | 34.7 [30.7;38.2] | <0.001 | 38.2 (6.34) | 35.8 (5.83) | 0.001 | 37.6 (5.66) | 35.0 (6.02) | <0.001 |
| immunoglobulin | 24.3 (4.79) | 22.6 (4.80) | <0.001 | 24.5 (4.77) | 23.5 (4.83) | 0.061 | 23.8 (4.34) | 23.5 (5.13) | 0.649 |
| DB | 4.60 [3.30;6.53] | 4.35 [3.20;5.93] | 0.071 | 4.70 [3.70;6.20] | 4.10 [3.05;5.80] | 0.014 | 4.35 [3.30;6.25] | 4.25 [3.38;6.00] | 0.853 |
| UDB | 7.10 [5.00;10.5] | 5.90 [4.07;9.25] | <0.001 | 8.20 [5.70;10.4] | 5.80 [4.15;7.75] | <0.001 | 6.70 [4.68;9.80] | 6.80 [4.50;9.10] | 0.403 |
| AKP | 71.0 [59.0;87.2] | 64.0 [52.0;81.0] | <0.001 | 71.0 [61.0;85.0] | 69.0 [54.5;84.0] | 0.252 | 70.0 [58.0;89.0] | 66.5 [53.8;82.0] | 0.140 |
| rGABA | 17.0 [12.0;25.0] | 14.0 [9.00;23.0] | <0.001 | 17.0 [12.0;25.0] | 15.0 [11.0;21.5] | 0.042 | 16.0 [11.0;24.0] | 14.0 [10.0;19.2] | 0.056 |
| ALT | 30.0 [17.0;54.0] | 31.5 [17.0;52.2] | 0.900 | 28.0 [16.0;55.0] | 30.0 [18.0;48.5] | 0.797 | 32.0 [16.0;54.2] | 31.0 [17.8;49.2] | 0.912 |
| AST | 30.0 [18.0;54.0] | 35.5 [19.0;60.2] | 0.021 | 28.0 [17.5;54.5] | 34.0 [19.0;53.0] | 0.362 | 30.0 [18.0;55.5] | 34.0 [19.8;64.0] | 0.324 |
| cholesterol | 4.19 [3.62;4.85] | 4.12 [3.55;4.70] | 0.078 | 4.23 (0.84) | 4.18 (0.89) | 0.625 | 4.13 [3.50;4.79] | 4.22 [3.60;4.68] | 0.729 |
| TBA | 2.85 [1.70;5.10] | 3.10 [1.80;6.55] | 0.049 | 3.00 [1.80;5.60] | 3.28 [2.00;7.42] | 0.088 | 2.95 [1.80;5.40] | 3.27 [2.00;5.65] | 0.312 |
| TG | 1.20 [0.89;1.62] | 1.12 [0.84;1.44] | 0.009 | 1.20 [0.94;1.67] | 1.14 [0.97;1.40] | 0.245 | 1.20 [0.90;1.69] | 1.14 [0.84;1.41] | 0.161 |
| HDL | 1.11 [0.96;1.31] | 1.15 [0.93;1.36] | 0.253 | 1.12 [0.98;1.31] | 1.15 [0.92;1.28] | 0.398 | 1.12 [0.94;1.32] | 1.12 [0.93;1.35] | 0.948 |
| LDL | 2.50 [2.08;2.93] | 2.39 [1.94;2.90] | 0.018 | 2.43 [2.10;2.91] | 2.45 [2.04;3.02] | 0.954 | 2.50 [1.97;2.91] | 2.50 [2.15;2.88] | 0.447 |
| Urea | 5.04 [4.20;5.99] | 5.01 [4.24;6.08] | 0.604 | 5.21 [4.08;6.08] | 4.86 [4.08;5.92] | 0.451 | 5.08 [4.20;6.04] | 5.07 [3.77;6.60] | 0.958 |
| uric_acid | 248 [204;301] | 234 [188;291] | 0.003 | 258 [204;310] | 223 [194;289] | 0.024 | 246 [200;293] | 238 [190;296] | 0.238 |
| Crine | 95.0 [85.0;106] | 92.0 [83.8;102] | 0.025 | 96.0 [85.0;106] | 97.0 [84.5;106] | 0.765 | 97.0 [86.0;106] | 91.0 [84.0;102] | 0.026 |
| GLU | 5.54 [4.99;7.25] | 5.82 [5.11;8.04] | 0.017 | 5.71 [5.10;7.00] | 5.64 [5.11;8.00] | 0.848 | 5.59 [5.07;7.09] | 5.72 [5.05;7.42] | 0.910 |
| Na | 141 [139;143] | 140 [138;142] | <0.001 | 141 [139;143] | 140 [138;142] | 0.001 | 141 (3.11) | 140 (3.09) | 0.213 |
| K | 4.13 (0.40) | 4.16 (0.42) | 0.307 | 4.14 (0.41) | 4.19 (0.37) | 0.204 | 4.11 (0.42) | 4.16 (0.45) | 0.303 |
| Cl | 105 [102;107] | 105 [102;107] | 0.939 | 105 [103;107] | 104 [101;106] | 0.077 | 104 [102;107] | 104 [102;107] | 0.811 |
| CysC | 0.78 [0.67;0.90] | 0.75 [0.62;0.89] | 0.072 | 0.78 [0.69;0.90] | 0.81 [0.69;0.93] | 0.441 | 0.80 [0.69;0.89] | 0.75 [0.62;0.83] | 0.007 |
| Ca2 | 2.07 [1.93;2.19] | 1.98 [1.88;2.10] | <0.001 | 2.08 (0.18) | 2.04 (0.15) | 0.062 | 2.06 (0.17) | 1.99 (0.17) | 0.002 |
| AFP | 2.72 [1.97;3.84] | 2.67 [1.95;4.35] | 0.521 | 2.83 [2.08;3.96] | 2.65 [1.98;3.90] | 0.494 | 2.49 [1.78;3.64] | 3.04 [2.13;4.71] | 0.001 |
| CEA | 2.04 [1.27;3.18] | 2.94 [1.72;6.33] | <0.001 | 2.07 [1.29;3.01] | 3.08 [1.92;11.1] | <0.001 | 1.96 [1.35;3.17] | 3.02 [2.09;13.8] | <0.001 |
| CA199 | 8.68 [5.45;14.7] | 34.2 [11.7;55.0] | <0.001 | 9.87 [5.46;16.6] | 35.5 [11.8;50.2] | <0.001 | 10.1 [6.21;16.2] | 32.2 [11.9;51.7] | <0.001 |
| CA125 | 10.6 [7.94;14.1] | 13.5 [9.15;20.8] | <0.001 | 10.7 [7.78;15.3] | 13.0 [8.34;20.9] | 0.007 | 11.0 [8.13;16.0] | 13.5 [9.66;23.2] | 0.001 |
| WBC | 7.84 [5.64;11.1] | 8.56 [5.91;11.6] | 0.195 | 7.39 [5.73;11.1] | 8.64 [6.00;11.4] | 0.380 | 7.39 [5.73;10.8] | 8.32 [6.73;11.3] | 0.201 |
| Neuro_R | 0.84 [0.63;0.91] | 0.88 [0.76;0.91] | <0.001 | 0.84 [0.62;0.90] | 0.87 [0.71;0.90] | 0.059 | 0.84 [0.63;0.90] | 0.87 [0.71;0.90] | 0.142 |
| Lymp_R | 0.13 [0.06;0.28] | 0.09 [0.06;0.16] | <0.001 | 0.12 [0.06;0.28] | 0.10 [0.06;0.19] | 0.072 | 0.12 [0.07;0.26] | 0.09 [0.06;0.20] | 0.028 |
| RBC | 4.38 (0.69) | 4.16 (0.63) | <0.001 | 4.36 [3.96;4.86] | 4.22 [3.81;4.74] | 0.080 | 4.38 (0.65) | 4.14 (0.56) | 0.001 |
| hemoglobin | 133 [115;148] | 118 [105;137] | <0.001 | 135 [112;150] | 123 [107;140] | 0.003 | 129 (23.5) | 118 (20.3) | <0.001 |
| HCT | 0.40 [0.35;0.44] | 0.37 [0.33;0.42] | <0.001 | 0.40 [0.35;0.45] | 0.38 [0.34;0.42] | 0.003 | 0.39 (0.06) | 0.37 (0.05) | <0.001 |
| PLT | 200 [156;246] | 192 [152;252] | 0.692 | 194 [159;250] | 208 [152;256] | 0.732 | 195 [157;240] | 207 [156;274] | 0.123 |
| D_2 | 300 [5.00;570] | 370 [158;900] | 0.002 | 270 [3.22;575] | 360 [145;1230] | 0.006 | 310 [130;782] | 370 [205;1185] | 0.026 |
| APTT | 34.6 [30.4;37.6] | 34.6 [31.5;38.2] | 0.319 | 34.2 [29.5;37.5] | 33.9 [30.5;38.0] | 0.350 | 35.0 [30.6;37.4] | 35.2 [32.2;38.8] | 0.140 |
| PT | 12.9 [12.0;13.5] | 13.2 [12.4;13.8] | 0.003 | 12.8 [11.9;13.5] | 13.0 [12.2;13.9] | 0.128 | 13.0 [12.1;13.7] | 13.2 [12.7;13.9] | 0.028 |
| SII | 1212 [448;2634] | 1802 [915;3005] | <0.001 | 1205 [512;2537] | 1817 [692;3026] | 0.029 | 1090 [563;2331] | 1759 [937;2891] | 0.003 |
| PNI | 38.0 [34.4;43.7] | 43.3 [37.6;50.4] | <0.001 | 39.6 [35.2;46.5] | 44.0 [37.8;50.7] | <0.001 | 38.6 [34.8;45.7] | 42.8 [38.5;49.5] | <0.001 |
| NLR | 6.65 [2.31;14.5] | 9.93 [4.96;15.3] | <0.001 | 6.81 [2.21;14.4] | 9.02 [3.79;14.2] | 0.085 | 7.02 [2.43;12.7] | 9.74 [3.68;14.2] | 0.035 |
| CCR | 120 [106;143] | 121 [105;146] | 0.790 | 118 [106;134] | 118 [100;140] | 0.978 | 124 [107;142] | 125 [110;141] | 0.615 |
| DB, Direct Bilirubin; UDB, Indirect Bilirubin; AKP, Alkaline Phosphatase; rGABA, Receptor for Gamma-Aminobutyric Acid; ALT, Alanine Aminotransferase; AST, Aspartate Aminotransferase; cholesterol, Total Cholesterol; TBA, Total Bile Acids; TG, Triglycerides; HDL, High-Density Lipoprotein; LDL, Low-Density Lipoprotein; Urea, Urea Nitrogen; uric_acid, Uric Acid; CysC, Cystatin C; GLU, Glucose; Na, Sodium; K, Potassium; Cl, Chloride; Ca2, Calcium; AFP, Alpha-fetoprotein; CEA, Carcinoembryonic Antigen; CA199, Carbohydrate Antigen 19-9; CA125, Carbohydrate Antigen 125; WBC, White Blood Cell Count; Neuro_R, Neutrophil Ratio; Lymp_R, Lymphocyte Ratio; RBC, Red Blood Cell Count; hemoglobin, Hemoglobin; HCT, Hematocrit; PLT, Platelet Count; D_2, D-dimer; APTT, Activated Partial Thromboplastin Time; PT, Prothrombin Time; SII, Systemic Immune-inflammation Index; PNI, Prognostic Nutritional Index; NLR, Neutrophil-to-Lymphocyte Ratio; CCR, Creatinine-to-Cystatin C Ratio. | | | | | | | | | |
