# Supplementary material for: From complex algorithms to clinical practice: a multicenter machine learning model and simplified decision tree for predicting cachexia risk in gastric cancer
Source: Front Oncol. 2026 Mar 10;16:1767547. doi: 10.3389/fonc.2026.1767547 (PMC13008652; doi:10.3389/fonc.2026.1767547)
Supplement: Supplementary file 3 [file Table2.docx]

| Table S2. Univariate logistic regression analysis of potential predictors of cachexia. | | | | |
| --- | --- | --- | --- | --- |
| **Characteristic** | **N** | **OR**^1^ | **95% CI**^1^ | **p-value** |
| gender | 1,570 |  |  |  |
| male |  | — | — |  |
| female |  | 1.16 | 0.90, 1.48 | 0.243 |
| age | 1,570 | 1.00 | 0.99, 1.01 | 0.586 |
| BMI | 1,570 | 0.72 | 0.69, 0.76 | **<0.001** |
| T stage | 1,570 |  |  |  |
| 1 |  | — | — |  |
| 2 |  | 2.82 | 1.59, 5.08 | **<0.001** |
| 3 |  | 6.40 | 3.96, 10.8 | **<0.001** |
| 4 |  | 26.2 | 16.8, 43.1 | **<0.001** |
| N stage | 1,570 |  |  |  |
| 0 |  | — | — |  |
| 1 |  | 1.97 | 1.36, 2.84 | **<0.001** |
| 2 |  | 4.72 | 3.34, 6.68 | **<0.001** |
| 3 |  | 10.0 | 7.48, 13.6 | **<0.001** |
| albumin | 1,570 | 0.92 | 0.90, 0.93 | **<0.001** |
| immunoglobulin | 1,570 | 0.95 | 0.93, 0.97 | **<0.001** |
| DB | 1,570 | 0.98 | 0.94, 1.01 | 0.204 |
| UDB | 1,570 | 0.94 | 0.91, 0.96 | **<0.001** |
| AKP | 1,570 | 1.00 | 0.99, 1.00 | 0.257 |
| rGABA | 1,570 | 1.00 | 1.00, 1.00 | 0.649 |
| ALT | 1,570 | 1.00 | 1.00, 1.00 | 0.435 |
| AST | 1,570 | 1.00 | 1.00, 1.00 | **0.027** |
| cholesterol | 1,570 | 0.92 | 0.81, 1.03 | 0.157 |
| TBA | 1,570 | 1.02 | 1.01, 1.04 | **<0.001** |
| TG | 1,570 | 0.85 | 0.72, 0.98 | **0.035** |
| HDL | 1,570 | 1.14 | 0.77, 1.69 | 0.514 |
| LDL | 1,570 | 0.91 | 0.78, 1.06 | 0.235 |
| Urea | 1,570 | 1.03 | 0.97, 1.10 | 0.302 |
| uric_acid | 1,570 | 1.00 | 1.00, 1.00 | **<0.001** |
| Crine | 1,570 | 0.99 | 0.99, 1.00 | **0.020** |
| GLU | 1,570 | 1.04 | 1.00, 1.09 | 0.064 |
| Na | 1,570 | 0.91 | 0.87, 0.94 | **<0.001** |
| K | 1,570 | 1.28 | 0.99, 1.67 | 0.064 |
| Cl | 1,570 | 0.99 | 0.96, 1.02 | 0.483 |
| CysC | 1,570 | 0.57 | 0.31, 1.01 | 0.056 |
| Ca2 | 1,570 | 0.12 | 0.06, 0.22 | **<0.001** |
| AFP | 1,570 | 1.01 | 1.01, 1.02 | **0.002** |
| CEA | 1,570 | 1.09 | 1.07, 1.11 | **<0.001** |
| CA199 | 1,570 | 1.05 | 1.05, 1.06 | **<0.001** |
| CA125 | 1,570 | 1.00 | 1.00, 1.00 | 0.092 |
| WBC | 1,570 | 1.02 | 0.99, 1.04 | 0.280 |
| Neuro_R | 1,570 | 9.49 | 4.42, 20.8 | **<0.001** |
| Lymp_R | 1,570 | 0.05 | 0.02, 0.12 | **<0.001** |
| RBC | 1,570 | 0.64 | 0.54, 0.75 | **<0.001** |
| hemoglobin | 1,570 | 0.98 | 0.98, 0.99 | **<0.001** |
| HCT | 1,570 | 0.00 | 0.00, 0.01 | **<0.001** |
| PLT | 1,570 | 1.00 | 1.00, 1.00 | 0.050 |
| D_2 | 1,570 | 1.00 | 1.00, 1.00 | **<0.001** |
| APTT | 1,570 | 1.02 | 1.00, 1.04 | **0.018** |
| PT | 1,570 | 1.17 | 1.08, 1.27 | **<0.001** |
| SII | 1,570 | 1.00 | 1.00, 1.00 | **<0.001** |
| PNI | 1,570 | 1.08 | 1.05, 1.09 | **<0.001** |
| NLR | 1,570 | 1.02 | 1.01, 1.03 | **<0.001** |
| CCR | 1,570 | 1.00 | 1.00, 1.00 | 0.571 |
| ^1^OR = Odds Ratio, CI = Confidence Interval, DB, Direct Bilirubin; UDB, Indirect Bilirubin; AKP, Alkaline Phosphatase; rGABA, Receptor for Gamma-Aminobutyric Acid; ALT, Alanine Aminotransferase; AST, Aspartate Aminotransferase; cholesterol, Total Cholesterol; TBA, Total Bile Acids; TG, Triglycerides; HDL, High-Density Lipoprotein; LDL, Low-Density Lipoprotein; Urea, Urea Nitrogen; uric_acid, Uric Acid; Crine, Creatinine; GLU, Glucose; Na, Sodium; K, Potassium; Cl, Chloride; CysC, Cystatin C; Ca2, Calcium; AFP, Alpha-fetoprotein; CEA, Carcinoembryonic Antigen; CA199, Carbohydrate Antigen 19-9; CA125, Carbohydrate Antigen 125; WBC, White Blood Cell Count; Neuro_R, Neutrophil Ratio; Lymp_R, Lymphocyte Ratio; RBC, Red Blood Cell Count; hemoglobin, Hemoglobin; HCT, Hematocrit; PLT, Platelet Count; D_2, D-dimer; APTT, Activated Partial Thromboplastin Time; PT, Prothrombin Time; SII, Systemic Immune-inflammation Index; PNI, Prognostic Nutritional Index; NLR, Neutrophil-to-Lymphocyte Ratio; CCR, Creatinine-to-Cystatin C Ratio. | | | | |
