# Supplementary material for: From complex algorithms to clinical practice: a multicenter machine learning model and simplified decision tree for predicting cachexia risk in gastric cancer
Source: Front Oncol. 2026 Mar 10;16:1767547. doi: 10.3389/fonc.2026.1767547 (PMC13008652; doi:10.3389/fonc.2026.1767547)
Supplement: Supplementary file 4 [file Table3.docx]

| Table S3. Multivariate logistic regression identifying independent predictors of cachexia. | | | |
| --- | --- | --- | --- |
| **Characteristic** | **OR**^1^ | **95% CI**^1^ | **P-value** |
| BMI | 0.69 | 0.64, 0.73 | **<0.001** |
| T stage |  |  |  |
| 1 | — | — |  |
| 2 | 1.81 | 0.92, 3.62 | 0.089 |
| 3 | 2.60 | 1.44, 4.85 | **0.002** |
| 4 | 8.48 | 4.70, 15.8 | **<0.001** |
| N stage |  |  |  |
| 0 | — | — |  |
| 1 | 0.79 | 0.48, 1.28 | 0.341 |
| 2 | 1.33 | 0.82, 2.18 | 0.249 |
| 3 | 1.86 | 1.19, 2.92 | **0.007** |
| albumin | 1.05 | 0.95, 1.16 | 0.361 |
| immunoglobulin | 0.97 | 0.93, 1.01 | 0.108 |
| UDB | 0.97 | 0.94, 1.01 | 0.113 |
| AST | 1.00 | 1.00, 1.00 | 0.962 |
| TBA | 1.01 | 0.99, 1.03 | 0.304 |
| TG | 1.07 | 0.87, 1.33 | 0.530 |
| Uric acid | 1.00 | 1.00, 1.00 | 0.082 |
| AFP | 1.01 | 1.01, 1.02 | **0.002** |
| CEA | 1.05 | 1.02, 1.08 | **<0.001** |
| CA199 | 1.04 | 1.03, 1.05 | **<0.001** |
| Na | 0.98 | 0.93, 1.04 | 0.556 |
| Lymp R | 0.24 | 0.01, 4.43 | 0.344 |
| RBC | 2.21 | 1.28, 3.84 | **0.005** |
| hemoglobin | 1.02 | 0.98, 1.05 | 0.340 |
| HCT | 0.00 | 0.00, 386 | 0.231 |
| Crine | 1.00 | 0.99, 1.01 | 0.960 |
| PLT | 1.00 | 1.00, 1.00 | 0.922 |
| D_2 | 1.00 | 1.00, 1.00 | 0.976 |
| PT | 1.15 | 1.0, 1.32 | 0.061 |
| SII | 1.00 | 1.00, 1.00 | 0.449 |
| PNI | 1.08 | 0.97, 1.17 | 0.173 |
| NLR | 0.97 | 0.92, 1.02 | 0.286 |
| ^1^OR = Odds Ratio, CI = Confidence Interval, UDB, Indirect Bilirubin; AST, Aspartate Aminotransferase; TBA, Total Bile Acids; TG, Triglycerides; uric acid, Uric Acid; AFP, Alpha-fetoprotein; CEA, Carcinoembryonic Antigen; CA199, Carbohydrate Antigen 19-9; Na, Sodium; Lymp R, Lymphocyte Ratio; RBC, Red Blood Cell Count; hemoglobin, Hemoglobin; HCT, Hematocrit; Crine, Creatinine; PLT, Platelet Count; D_2, D-dimer; PT, Prothrombin Time; SII, Systemic Immune-inflammation Index; PNI, Prognostic Nutritional Index; NLR, Neutrophil-to-Lymphocyte Ratio. | | | |
