# Supplementary material for: From complex algorithms to clinical practice: a multicenter machine learning model and simplified decision tree for predicting cachexia risk in gastric cancer
Source: Front Oncol. 2026 Mar 10;16:1767547. doi: 10.3389/fonc.2026.1767547 (PMC13008652; doi:10.3389/fonc.2026.1767547)
Supplement: Supplementary file 5 [file Table4.docx]

Table S4. Variance Inflation Factors (VIFs) for predictors of cachexia.

| Characteristics | GVIF | | Df | GVIF^(1/(2*Df)) |
| --- | --- | --- | --- | --- |
| BMI | | 1.243172 | 1 | 1.114976 |
| operation | | 1.138417 | 1 | 1.066966 |
| T | | 1.596065 | 3 | 1.08104 |
| N | | 1.602548 | 3 | 1.081771 |
| M | | 1.043302 | 1 | 1.021422 |
| albumin | | 14.40884 | 1 | 3.795898 |
| immunoglobulin | | 1.684522 | 1 | 1.297891 |
| UDB | | 1.16728 | 1 | 1.080407 |
| AST | | 1.351875 | 1 | 1.162702 |
| TBA | | 1.05712 | 1 | 1.028163 |
| TG | | 1.130936 | 1 | 1.063455 |
| Uric acid | | 1.426013 | 1 | 1.194158 |
| AFP | | 1.114741 | 1 | 1.055813 |
| CEA | | 1.097211 | 1 | 1.047479 |
| CA199 | | 1.148598 | 1 | 1.071727 |
| Na | | 1.151589 | 1 | 1.073121 |
| Lymph Ratio | | 4.298472 | 1 | 2.073276 |
| RBC | | 5.184244 | 1 | 2.276893 |
| hemoglobin | | 26.71173 | 1 | 5.16834 |
| HCT | | 36.96937 | 1 | 6.080245 |
| Crine | | 1.536793 | 1 | 1.239674 |
| PLT | | 3.100258 | 1 | 1.760755 |
| D_2 | | 1.316897 | 1 | 1.147561 |
| PT | | 1.34499 | 1 | 1.159737 |
| SII | | 10.3926 | 1 | 3.223756 |
| PNI | | 21.90709 | 1 | 4.680501 |
| NLR | | 10.09588 | 1 | 3.177401 |
| CCR | | 1.487317 | 1 | 1.219556 |
